# Methods of induction and augmentation of labor in a freestanding birth center: a cross-sectional study

**DOI:** 10.1590/1980-220X-REEUSP-2023-0158en

**Published:** 2024-01-19

**Authors:** Gisele Almeida Lopes, Thais Trevisan Teixeira, Nathalie Leister, Maria Luiza Riesco

**Affiliations:** 1 Universidade de São Paulo, Escola de Enfermagem, Programa de Pós-Graduação em Enfermagem, São Paulo, SP, Brazil.; 2 Centro de Parto Humanizado Casa Angela, São Paulo, SP, Brazil.; 3 City, University of London, School of Health & Psychological Sciences, Centre for Maternal & Child Health Research, London, United Kingdom.; 4 Universidade de São Paulo, Escola de Enfermagem, Departamento de Enfermagem Materno-Infantil e Psiquiátrica, São Paulo, SP, Brazil.

**Keywords:** Birthing Centers, Complementary Therapies, Parturition, Centros de Asistencia al Embarazo y al Parto, Terapias Complementarias, Parto, Centros de Assistência à Gravidez e ao Parto, Terapias complementares; Parto

## Abstract

**Objective:**

To describe and analyze the use of methods of induction and augmentation of labor in a freestanding birth center (FBC).

**Method:**

Cross-sectional study carried out at a FBC located in São Paulo (SP), with all women booked from 2011 to 2021 (n = 3,397).

**Results:**

The majority of women (61.3%) did not receive any method. The methods were used alone or in combination (traditional Chinese medicine, massage, castor oil, stimulating tea, amniotomy, and oxytocin). Traditional Chinese medicine (acupuncture, acupressure, and moxa) was the most used method (14.7%) and oxytocin was the least frequent (5.1%). The longer the water breaking time, the greater the number of methods used (p < 0.001). Amniotomy was associated with maternal transfers (p < 0.001).

**Conclusion:**

Induction and augmentation of labor were strictly adopted. The use of natural or non-pharmacological methods prevailed. Robust clinical studies are needed to prove the effectiveness of non-pharmacological methods of stimulation of childbirth, in addition to strategies for their implementation in other childbirth care services, to really prove the effectiveness of non-pharmacological methods in the parturition process, that is, in labor and birth.

## RESUMO

**Objetivo:**

Descrever e analisar o uso de métodos de indução e condução do parto em centro de parto normal peri-hospitalar (CPNp).

**Método:**

Estudo transversal realizado em um CPNp localizado em São Paulo (SP), com a totalidade das mulheres atendidas de 2011 a 2021 (n = 3.397).

**Resultados:**

A maioria das mulheres (61,3%) não recebeu qualquer método. Os métodos foram utilizados isoladamente ou de forma combinada (medicina tradicional chinesa, massagem, óleo de rícino, chá estimulante, amniotomia e ocitocina). A medicina tradicional chinesa (acupuntura, acupressão e moxa) foi o método mais usado (14,7%) e a ocitocina foi o menos frequente (5,1%). Quanto maior o tempo de bolsa rota, maior o número de métodos utilizados (p < 0,001). A amniotomia esteve associada às transferências maternas (p < 0,001).

**Conclusão:**

A indução e condução do parto foram adotadas de forma restrita. Prevaleceu o uso dos métodos naturais ou não-farmacológicos. São necessários estudos clínicos robustos para comprovar a eficácia dos métodos não farmacológicos de estímulo do parto, além de estratégias para sua implementação em outros serviços de assistência ao parto, para realmente comprovar a eficácia de métodos não farmacológicos no processo de parturição, isto é: no trabalho de parto e nascimento.

## RESUMEN

**Objetivo:**

Describir y analizar el uso de métodos de inducción y manejo del parto en un centro de parto normal perihospitalario (CPNp).

**Método:**

Estudio transversal realizado en un CPNp ubicado en São Paulo (SP), con todas las mujeres atendidas entre 2011 y 2021 (n = 3.397).

**Resultados:**

La mayoría de las mujeres (61,3%) no recibió ningún método. Los métodos se utilizaron solos o en combinación (medicina tradicional china, masajes, aceite de ricino, té estimulante, amniotomía y oxitocina). La medicina tradicional china (acupuntura, acupresión y moxa) fue el método más utilizado (14,7%) y la oxitocina el menos frecuente (5,1%). Cuanto mayor es el tiempo de rotura de la bolsa, mayor es el número de métodos utilizados (p < 0,001). La amniotomía se asoció con transferencias maternas (p < 0,001).

**Conclusión:**

La inducción y manejo del parto se adoptaron de forma restringida. Predominó el uso de métodos naturales o no farmacológicos. Se necesitan estudios clínicos sólidos para demostrar la eficacia de los métodos no farmacológicos de estimulación del parto, además de estrategias para su implementación en otros servicios de atención al parto, para comprobar realmente la efectividad de los métodos no farmacológicos en el proceso del parto, es decir: en el trabajo de parto y el nacimiento.

## INTRODUCTION

It is the pregnant woman’s right to know and choose the place where her pregnancy, birth and postpartum will be cared, according to her gestational risk. Freestanding birth centers (FBC), also called birth houses, are safe places to care for pregnant women with straightforward pregnancies^(1)^.

FBC’s main focus is on holistic health care for pregnant women and newborns from the gestational period to the postpartum and neonatal period. One of the criteria for care in these places is that the pregnant woman has a straightforward pregnancy. Given that the classification of risk is multifactorial and that there may be changes in the degree of risk throughout pregnancy, there are divergences in the criteria for monitoring pregnant women, depending on the care protocol of each FBC. The services teams are led by midwives trained to attend to childbirth and emergencies, as well as carry out transfers if the woman or newborn requires hospital care^(2)^.

The Brazilian Ministry of Health (MS) recommends supporting women’s decision to have care in a birth center, as well as supporting the use of non-pharmacological approaches during labor and reducing routine invasive procedures, such as amniotomy and early use of oxytocin^(3)^. In this regard, the World Health Organization (WHO) support the use of relaxation and breathing techniques, music, mindfulness, massage, and freedom of position among other non-invasive techniques for pain relief according to the pregnant woman’s wishes^(4)^.

Since 2006, the Brazilian government has encouraged the adoption of Complementary and Integrative Health Practices (CIHP), through the National Policy on Integrative and Complementary Practices in the Brazilian Public Health System (SUS), which was updated in 2018, with the incorporation of new practices^(5)^. CIHP are non-pharmacological practices with broad possibilities for action in health care. During childbirth, they are used for relaxation, pain relief, reduction of anxiety and also to stimulate the start and augmentation of labor.

The procedure used to stimulate the onset of labor, if the spontaneous onset does not occur, is called induction. It is indicated when there are maternal or fetal conditions warranting it. Unlike induction, labor augmentation consists of the use of practices that accelerate the slow or ineffective course of labor that has already started spontaneously^(6,7)^.

Usually, the diagnosis of labor is made based on the pregnant woman’s complaints and with a complete obstetric examination, including vaginal examination to assess the consistency and dilation of the cervix and the thickness of the cervical canal and the assessment of uterine dynamics (UD). This assessment is essential and is measured by the frequency, length, and intensity of contractions within a 10-minute interval. During the examination, the professional rests his hand flat on the woman’s abdomen for a period of 10 minutes and assesses the UD, perceived mainly in the uterine fundus^(8)^.

The presence of two or more contractions within 10 minutes with an average duration of 50 to 60 seconds and uterine stiffening concomitant with painful cramps are considered effective uterine contractions. During pregnancy, the woman can have contractions of different frequencies, length, and intensity. At term, prodromes or the premonitory period of labor, contractions intensify without producing a significant increase in cervical dilation. Physiologically, this condition will progress to labor^(6,7)^.

The first stage of labor, or dilation phase, begins with rhythmic contractions that progressively modify and dilate the cervix, reaching full dilation. This stage is divided into the latent and active phases of labor. The latent phase corresponds to less frequent uterine contractions, with slow dilation and modification of the cervix, lasting around 20 hours. The active phase is characterized by faster cervical dilation, with a variable average duration^(6,7)^.

The second stage of labor, or pushing, occurs when there is total dilation of the cervix and ends when the baby is born. Contractions tend to be more intense and frequent, reaching up 5 contractions of 60 seconds in duration, every 10 minutes. In the third stage, complete placental delivery occurs; finally, the fourth stage of labor, or Greenberg’s period, is the moment when uterine contraction effectively occurs, together with thrombotamponage and myotamponage, lasting between one and two hours after birth^(6,7,9)^.

In addition to the clinical conditions that may indicate the need to induce labor, several factors can influence its physiological progression. The causes may be related to decreased UD, fetal malpositioning, intense pain, among others. Pharmacological and non-pharmacological methods can help to correct these clinical conditions. Regarding CIHP used to facilitate the onset of labor, studies present large gaps and heterogeneity, making it difficult to adequately assess its effectiveness^(2)^.

Methods such as the consumption of evening primrose oil, dates, teas, acupressure, massages, and acupuncture are documented. However, there is a lack of well-designed studies that confirm strong scientific evidence about the effectiveness of these methods. The consumption of evening primrose oil, for example, differs in relation to its effectiveness in cervical maturity, in two different systematic reviews^(10,11)^. Some studies have shown that the consumption of dates, within a specific dosage and period, contributed to reducing the active phase of labor and cervical ripening^(12)^.

Just like the use of dates, acupressure, at point SP6, was also related to a shorter period of active phase^(13)^. In general, CIHP are less invasive and generally lower-cost methods, which can help when there is a need to induce or augment the labor. The oral use of castor oil, for example, has been shown to be effective in inducing labor in specific scenarios, being a low-cost and easy-to-use method, so it can be considered in cases where the labor onset is required^(14,15)^. Another method is the membrane detachment, which when used, reduces the need for pharmacological induction when compared to expectant management^(16)^. Other methods used empirically, such as teas, specific massages and others, requires studies that analyze their use, risks, and benefits.

In São Paulo city, FBC are guided by the Technical Manual for Freestanding Birth Centers of the Municipality of São Paulo^(17)^, which recommends the use of CIHP. Together with different non-pharmacological methods, its use in labor has several indications, such as relieving pain, promoting comfort and relaxation, helping with fetal descent, rotation and oxygenation. Consequently, even without specific indication for induction or augmentation of labor, some methods favor this outcome, as a secondary effect.

Aiming to evaluate the use of methods for induction and augmentation of labor, the aim of this study was to describe and analyze the use of methods for induction and augmentation of labor in a FBC.

## METHOD

### Study Design

Cross-sectional study.

### Local

Study carried out in a FBC located in the south zone of the city of São Paulo, Brazil. This birth center provides prenatal, childbirth and postnatal care and, since 2016, has maintained an agreement with the City Hall of São Paulo and receives financial resources from SUS.

The service is provided exclusively to women with straightforward pregnancies who meet the inclusion criteria for care during prenatal and childbirth care, described in the Technical Manual for Freestanding Birth Centers of the Municipality of São Paulo^(17)^.

Prenatal and childbirth care is provided by midwives with the support of auxiliary nurses. During labor and after birth, the woman and baby can be transferred in case of clinical or obstetric reasons that indicate the need for care in a hospital environment; or if there is a desire expressed by the pregnant woman. The transfer are made by the institution’s own ambulance, to the referral hospital or another hospital of the woman’s choice.

### Study Variables

The variables studied were the methods of inducing and augmenting labor described in the aforementioned Technical Manual^(17)^ and adopted at the study site. They are used to induce labor during prenatal consultations, from 40 weeks of gestation, or in cases of admission to the FBC due to premature rupture of membranes (PROM), or even when there is an indication to augment labor. These methods are presented below.

Stimulant tea is used with the aim of stimulating UD. It can be used in women with 40 gestational weeks or more, women in labor with ineffective uterine dynamics, PROM, in the latent phase or with painful prodromes. The tea ingredients are cinnamon, cloves, ginger, verbena, cocoa powder, and black pepper.

Castor oil is also used to stimulate UD from 40 gestational weeks and in cases of PROM. It can be prepared in two ways: in an oil shake mixed with fruit, or by adding the oil to a fried egg; in both recipes, a measure of 1 to 2 tablespoons of Castor oil is used. Side effects that may occur are diarrhea and vomiting.

Abdominal massage with stimulating clove, cinnamon and ginger oil is a method used to stimulate contractions. To prepare the stimulating oil, one drop of each clove, cinnamon, and ginger essential oil is used for 10 ml of base oil, such as grape seed, for example. After placing a small amount of the prepared oil on the hands and spreading it, massage on the abdomen with both hands starts. It is based on the rhythmic massage of anthroposophic medicine, which consists of performing a light glide, with circular movements. On the side of the fetal back, a Half-moon movement is made, and on the other side, a circular movement, like the Sun. The massage is performed for approximately 5 minutes. It can be used in women from 40 gestational weeks, in prodromes, painful latent phase, or with PROM^(17)^.

The Technical Manual^(17)^, used as a care protocol at the study site, also describes the application of acupuncture and acupressure. In this study, these two methods and moxa are called traditional Chinese medicine (TCM) methods. The acupuncture application protocol was created by professionals with postgraduate diploma in acupuncture. As it is easy to apply and has minimal side effects, it is used by the entire care team after training. The aim of applying TCM is to stimulate specific points on the woman’s body to trigger contractions or augment the labor.

In the FBC studied, in addition to these natural methods described, the administration of oxytocin and amniotomy (artificial rupture of ovular membranes) is indicated to augment labor or stimulate the progression of labor. According to the Technical Manual^(17)^, amniotomy should be avoided and used with caution, only when it is clearly beneficial in cases of functional dystocia and doubtful amnioscopy, and is contraindicated in cases of high fetal presentation, due to the risk of cord prolapse. Oxytocin, in its turn, is also used cautiously, in cases of stoppage of labor progression in the active phase or third stage of labor.

### Population and Selection Criteria

For this study, all women admitted for childbirth at the study site were included, from 01/01/2012 to 31/12/2021.

### Data Collection

Data collection happened between July 2017 and January 2023, through the analysis of women’s records and FBC admission, birth, and transfer books, using as instrument, a form developed by the researchers, with information related to maternal characteristics, labor and birth care, as well as data on the postnatal period during woman and baby stay.

### Data Analysis

The natural or non-pharmacological methods of inducing and augmenting labor (stimulating tea, castor oil, massage and TCM) were analyzed exclusively in a descriptive way. For amniotomy and oxytocin, the association with water breaking length, postpartum hemorrhage (PPH) and maternal transfer (intra or postpartum) was also analyzed.

Data were analyzed using absolute and relative frequencies for categorical variables. For bivariate analysis of the association between exposure variables and outcomes, the chi-square test and Kendall’s tau correlation coefficient were used. The type I error adopted was 5%.

### Ethical Aspects

This study was approved by the Ethics Committee of the School of Nursing of the University of São Paulo (CEP-EEUSP) (Opinion no. 2.026.648; 2017) and authorized by the Institution’s Scientific Committee. The informed consent form was waived by the Ethics Committee since data collection was carried out through the institution’s records and birth books.

## RESULTS

The total number of women admitted between January 1, 2012 and December 31, 2021 was 3,424. Those whose records were not located (n = 5) and those who gave birth on their way to the FBC (n = 22) were excluded, totaling for this study 3,397 women.

The women’s ages ranged from 14 to 45 years old, and the majority (86.4%) were between 20 and 35 years old. Over half declared themselves white (57.1%), followed by brown or black (39%). Most of them had finished high school or higher education (45% and 47.2% respectively); furthermore, most were nulliparous (72.4%) (Table 1).

**Table 1:** Distribution of women according to sociodemographic characteristics and parity – São Paulo, SP, Brazil, 2012–2021.

Variable	n	%
**Age**	**3397**	
20	192	5.7%
≥ 20 ≤ 35	2936	86.4%
> 35	269	7.9%
**Skin color**	**2788**	
White	1591	57.1%
Brown	780	28.0%
Black	307	11.0%
Yellow	85	3.0%
Indigenous	25	0.9%
**Level of education**	**2854**	
High education	1346	47.2%
High school	1285	45.0%
Elementary school	162	5.7%
Unfinished elementary school	61	2.1%
**Parity**	**3397**	
No previous birth	2460	72.4%
With previous birth	937	27.6%

Regarding the methods of inducing and augmenting labor, 61.3% (n = 2,081) of women did not use any of the methods, pharmacological or non-pharmacological, analyzed in this study. The most used method was TCM, used by 14.7% of women, followed by amniotomy (12.5%). The least used method was oxytocin (5.1%). Some women (11.8%) used more than one natural method to induce or augment the labor (Table 2).

**Table 2:** Distribution of women according to the induction and augmentation methods used – São Paulo, SP, Brazil, 2012–2021.

Variable	n	%
**Oxytocin**	**3397**	
Yes	174	5.1%
No	3223	94.9%
**Amniotomy**	**3397**	
Yes	425	12.5%
No	2972	87.5%
**Traditional Chinese Medicine**	**3397**	
Yes	501	14.7%
No	2896	85.3%
**Massage**	**3397**	
Yes	401	11.8%
No	2996	88.2%
**Castor oil**	**3397**	
Yes	373	11.0%
No	3024	89.0%
**Stimulating tea**	**3397**	
Yes	328	9.7%
No	3069	90.3%
**Use of natural methods**	**3397**	
No method	2449	72.1%
1 method	547	16.1%
2 methods	207	6.1%
3 methods	134	3.9%
4 methods	60	1.8%

**Figure 1:**
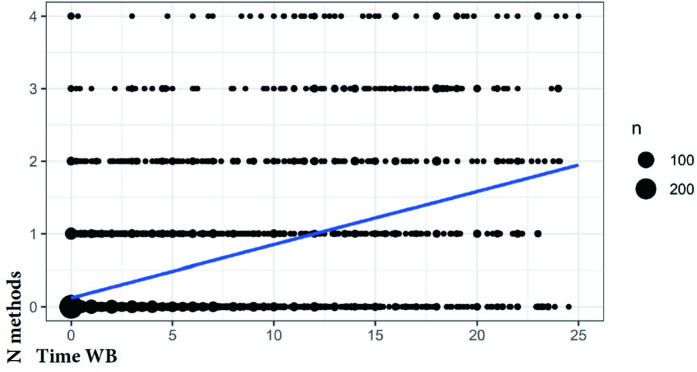
Distribution of women according to length of water breaking and use of non-pharmacological methods of labor induction and augmentation, São Paulo, SP, Brazil, 2012–2021. N.methods: number of methods; n: number of women (in proportion); Time WB: water breaking time; Kendall’s tau correlation coefficient (p < 0.001).

Regarding the integrity of the amniotic sac, Figure 1 shows that the longer the time of water breaking, the greater the number of natural methods used during labor (p < 0.001).

In more than half of women (54%), oxytocin was used in combination with another method of inducing or augmenting labor. Oxytocin was mainly combined with natural methods (Table 3).

**Table 3:** Distribution of women according to the use of oxytocin combined or not with other induction and conduction methods – São Paulo, SP, Brazil, 2012–2021.

Variables	174	%
Oxytocin	80	46.1%
Oxytocin + CIHP (stimulant tea, massage, castor, TCM)	62	35.6%
Oxytocin + amniotomy	22	12.6%
Oxytocin + amniotomy + CIHP (stimulating tea, massage, castor oil, TCM)	10	5.7%

Of the total number of women in the study, PPH occurred in 7.3% (n = 248) (data not shown in the table) and intrapartum and postpartum maternal transfers occurred in 19.2% (n = 652) and 2.6% (n = 87) of them, respectively (data not shown in the table). Table 4 shows these outcomes among women who underwent amniotomy or received oxytocin. Amniotomy was associated with transfers (p < 0.001); regarding the use of oxytocin, there was no significant difference between PPH and maternal transfer outcomes.

**Table 4:** Association between the use of amniotomy or oxytocin and postpartum hemorrhage and maternal transfer – São Paulo, SP, Brazil, 2012– 2021.

Variable	Amniotomy		p-value*	Oxytocin		p-value*
	Yes	No		Yes	No	
**Postpartum hemorrhage**						
Yes	34 (8.0%)	215 (7.2%)	0.571	9 (5.2%)	240 (7.5%)	0,262
No	391 (92.0%)	2757 (92.3%)		165 (94.8%)	2983 (92.5%)	
**Transfer**						
No	297 (69.9%)	2362 (79.5%)		127 (73.0%)	2532 (78.5%)	
Intrapartum	116 (27.3%)	535 (18.0%)	**<0.001**	41 (23.6%)	610 (19.0%)	0.216
Postpartum	12 (2.8%)	75 (2.5%)		6 (3.4%)	81 (2.5%)	

*Chi-square test.

## DISCUSSION

Common practices in FBC, such as admission in the active phase of labor, freedom of movement, exercise, and the use of non-pharmacological methods for pain relief can help to reduce the need for interventions during childbirth and the use of augmentation and induction methods of labor^(18)^. In this study, it was found that 61.3% of women did not use any pharmacological or non-pharmacological method of inducing or augmenting labor, indicating that even natural methods are used judiciously in FBC and that, in most cases, the birth happens naturally and physiologically, which may be related to good birth care practices, which is a characteristic of the place^(19)^.

The literature shows that there may be some improvement in cervical maturity and stimulation of uterine contractions when methods such as acupuncture, acupressure and moxa are used^(20,21)^. In this study, the most used non-pharmacological method of inducing and augmenting labor was TCM, with these methods being used more often and chosen as the first option among natural methods maybe because they cause little or no adverse effects. Acupressure and moxa techniques are easy to apply and even professionals without additional qualification can apply them after training. Furthermore, the team has a large number of professionals with additional training in acupuncture, another possible reason why TCM is used by the team of midwives on site.

In this study, 11% of women used castor oil to induce labor. Castor oil is considered by the Brazilian Health Regulatory Agency-Anvisa to be a laxative, and the literature suggests that it may promote cervical ripening and uterine contractions through the activating effect of the metabolite ricinoleic acid on prostaglandin EP3 receptors in the smooth muscles of the uterus and intestine^(22)^. Although the use of castor oil can cause some adverse effects such as nausea, vomiting, and diarrhea, serious adverse effects have not been demonstrated. Furthermore, it is demonstrated in the literature as an effective, low-cost, and non-harmful method for inducing labor^(14,15)^.

The use of integrative and complementary practices based on scientific evidence is part of the study site routine and is a characteristic of the care provided there, as well as empirical and holistic care based on the anthroposophic medicine. In this sense, although this study shows that the care team offers stimulating tea to women, that there is a description of its use in another birth center protocol^(23)^, and the report of the experience of midwives who used the technique and did not observe any adverse effects^(24)^, there is no evidence in the literature that corroborates the use of this method to stimulate labor.

Likewise, there is no scientific evidence of the use of stimulating massage to induce or augment labor. However, the technique followed is based on rhythmic massage, founded on the principles of anthroposophic medicine and studied in the literature in other areas of health care. In childbirth care, the aim of this massage is to align the neurosensory, rhythmic, and metabolic-motor systems and the physical, ether, and astral body of the human being to stimulate the release of endorphins and endogenous oxytocins that naturally stimulate labor^(25)^. An example is the randomized controlled study that showed specific stimulation in the autonomic nervous system in people who received rhythmic massage^(26)^.

As the care protocol suggests, after 18 or 24 hours of water breaking, depending on the results of the group B *Streptococcus agalactiae* (GBS), the parturient must be transferred to the hospital^(17)^. Therefore, non-pharmacological practices to stimulate uterine activity are started more quickly in women with PROM and no active labor. As shown in this study, the longer the time of water breaking, more methods are used in an attempt to make the birth happen within the time limit for transfer to the hospital, avoiding the need for pharmacological induction in the hospital.

The use of oxytocin in FBC is only recommended in cases where labor progression has stopped, that is, it should only be used as a way of augmentation, when the woman is already in the active phase of labor^(17)^. The use of this intervention early and without indication is not recommended by the WHO or the Brazilian Ministry of Health, as there is no clear evidence in the literature that the benefits outweigh the potential risks of tachysystole, fetal heart rate abnormalities, uterine rupture, water intoxication, and adverse cardiovascular effects^(3,4)^.

In this study, oxytocin was used judiciously, in only 5.1% of women. The percentage of oxytocin use was much lower than in other studies carried out in FBC in Brazil, where the use ranged from 27.5% to 34.8% of births^(27,28–30)^. Furthermore, in 55.2% of women who used oxytocin, the practice was combined with amniotomy or natural methods, demonstrating that in most cases, oxytocin is not the main method of choice for augmenting labor in the FBC.

Amniotomy to prevent delay in labor is not recommended. It should be considered when failure to labor progress is doubt. Furthermore, it is important that the woman is informed about the reason for carrying out the procedure and the possible increase in the uterine contractions intensity^(3)^. In this study, amniotomy was performed in 12.5% of women, also in a judicious manner. Its performance was less frequent when compared to studies carried out in other FBC in Brazil, where the rate varied from 30.6% to 67.6%^(27,29)^.

Even though birth in FBC is associated with lower rates of unnecessary interventions, it is worth considering that this variation in rates of oxytocin use^(27–30)^ and aminotomy^(27,29)^, shown in the literature, certainly reflects the differences in the care protocols followed. The little use of these practices in the FBC studied certainly reflects the adoption of CIHP and the recommendations of the Technical Manual for Freestanding Birth Centers in the Municipality of São Paulo^(17)^.

The comparison of use of oxytocin and amniotomy to PPH outcomes and maternal transfers shows the association of amniotomy with intrapartum maternal transfers. There was no significant difference between the association of the use of oxytocin and the outcomes studied. One of the factors that may be related to amniotomy and a greater chance of intrapartum transfer is the presence of meconium found after the procedure, as according to the institution’s protocol, when meconium is present, the woman must be transferred to the hospital^(17)^.

Considering the adverse effects caused by pharmacological methods of stimulating labor, including abnormal pattern of uterine contractions and neonatal complications, and the favorable effects of non-pharmacological methods, the latter can be considered effective and low-cost methods for inducing and augmenting labor when compared to the former^(11)^. However, unlike the reality of other health services, in the FBC studied, natural methods are disseminated and used by the team with support from the institutional protocol that describes their forms of use, being a possible facilitator for the team to make wide use of these methods. Therefore, the creation and implementation of protocols to expand the use of natural methods in health institutions, as well as further research in the literature on the effectiveness of these methods for stimulating labor, are required.

By contributing to the characterization of the care provided at the FBC, we expect to provide subsidies for the implementation of protocols for non-pharmacological induction and augmentation of labor, expanding the use of CIHP and the autonomy of midwives to indicate and promote its use.

The limitations of this research are related to the study’s data source, which is a database fed with information from the records of women and babies cared at the FBC. In this sense, when more than one method was adopted, it was not possible to reliably determine the sequence of its use in each labouring woman. Although completion of records is well qualified by the institution, the results are subject to information bias due to incorrect or missing data.

## CONCLUSION

The methods of inducing and augmenting labor were used sparingly at the FBC, and the vast majority of births occurred physiologically.

The most used method was TCM, the least used was oxytocin, and non-pharmacological methods prevailed in childbirth care in the studied FBC; furthermore, when oxytocin was used, it was most often combined with another method.

The use of a greater number of methods was associated with the length of water breaking, demonstrating the practice of contractions stimulation in cases of PROM, in line with the institutional protocol.

Regarding the maternal outcomes studied, oxytocin and amniotomy were not associated with PPH, but amniotomy was associated with maternal transfer.

The methods studied are described and supported by the institutional protocol, which provides wide adoption by the team of midwives. However, robust clinical studies are needed to prove the effectiveness of natural methods of labor stimulation, as well as strategies for their implementation and use in other childbirth care services.
